# Anterograde trafficking signals in GABA_A_ subunits are required for functional expression

**DOI:** 10.1080/19336950.2019.1676368

**Published:** 2019-10-15

**Authors:** Jessica L. Nuwer, Mark W. Fleck

**Affiliations:** Neuroscience & Experimental Therapeutics, Albany Medical College, Albany, NY, USA

**Keywords:** GABAA, trafficking, beta subunit, theta subunit, chimera

## Abstract

Pentameric GABA_A_ receptors are composed from 19 possible subunits. The GABA_A_ β subunit is unique because the β_1_ and β_3_ subunits can assemble and traffic to the cell surface as homomers, whereas most of the other subunits, including β_2_, are heteromers. The intracellular domain (ICD) of the GABA_A_ subunits has been implicated in targeting and clustering GABA_A_ receptors at the plasma membrane. Here, we sought to test whether and how the ICD is involved in functional expression of the β_3_ subunit. Since θ is the most homologous to β but does not form homomers, we created two reciprocal chimeric subunits, swapping the ICD between the β_3_ and θ subunits, and expressed them in HEK293 cells. Surface expression was detected with immunofluorescence and functional expression was quantified using whole-cell patch-clamp recording with fast perfusion. Results indicate that, unlike β_3_, neither the β_3_/θ_IC_ nor the θ/β_3IC_ chimera can traffic to the plasma membrane when expressed alone; however, when expressed in combination with either wild-type α_3_ or β_3_, the β_3_/θ_IC_ chimera was functionally expressed. This suggests that the ICD of α_3_ and β_3_ each contain essential anterograde trafficking signals that are required to overcome ER retention of assembled GABA_A_ homo- or heteropentamers.

## Introduction

Epilepsy, anxiety, neurodevelopmental disorders, and neuropsychiatric disorders collectively affect a significant proportion of the population. One common problem in these disorders is the dysfunction of the GABA_A_ receptor, so it is important to understand how these receptors are functionally regulated. GABA_A_ receptors are the target of anesthetics as well as drugs that are used as anticonvulsives, anxiolytics, and hypnotics [1]. They are pentameric ligand-gated chloride channels in the Cys-Loop superfamily of ligand-gated ion channels. Their role is to mediate fast inhibitory neuronal transmission. The pentameric ion channel is formed from a pool of 19 different GABA_A_ subunits (α_1-6_, β_1-3_, γ_1-3_, δ, ϵ, θ, π, and ρ_1-3_) [1], with each subunit having the same general structure: a long N-terminal domain (NTD) which creates the extracellular ligand binding domain (LBD), 4 transmembrane domains (TMD) that create the ion channel pore, a variable length intracellular domain (ICD) composed of the TM3-TM4 loop, and a short extracellular C-terminus (CTD) [1,2]. Considering the vast number of possible subunit combinations that could exist, it is important to understand the cellular mechanisms that limit the functional expression (assembly and trafficking) of subunit combinations that might otherwise exist. Most of the known combinations require α and β subunits; however, the rules that govern assembly and regulate trafficking of the receptors are still poorly understood.

Canonical GABA_A_ receptors contain 2 α subunits, 2 β subunits, and a third X subunit arranged counterclockwise around the central pore in the order: β-α-β-α-X; where X is typically a γ subunit; however, it is generally accepted that α, β, δ, or one of the other subunits can replace the γ [1]. Some of the structural elements that are involved in regulated assembly of compatible subunits have been identified, most commonly in the NTD binding loops that form the subunit-subunit interface [3–7]. Assembly of the β subunit is unique in that the β_1_ and β_3_ subunits can assemble and traffic to the plasma membrane as homomeric receptors, whereas most of the other subunits, including β_2_, are obligatory heteromers [8]. β_1_, β_2_ and β_3_ are at minimum 70% homologous but show differences in homomeric assembly. The critical determinant of β_3_ homomeric functional expression was identified as the so-called GKER sequence, located in the extracellular domain (ECD) binding loop F of β_3_, which corresponds to a DNTK sequence at the equivalent site in β_2_[7]. Using chimeric exchanges between β_2_ and β_3_, Taylor et. al. (1999) [7] showed that the GKER sequence was necessary for β_3_ and sufficient to rescue β_2_. This study implied that the determinants of functional expression are entirely contained in the NTD; however, there may be other determinants of functional expression that were not revealed in that study because they are sufficiently conserved across the β subunits. The TM3-TM4 intracellular loop (intracellular domain, ICD) has been implicated in targeting, anchoring, and clustering of the GABA_A_ receptor at the plasma membrane; however, the mechanism of how this loop is involved in GABA_A_ receptor functional expression is still largely unknown.

Here, we sought to test whether and how the ICD is involved in functional expression of the β_3_ subunit. We created two reciprocal chimeric subunits, one having the EC and TM domains from β_3_ and the ICD from θ, and the other having the EC and TM domains from θ and the ICD from β_3_. The θ subunit was chosen as the chimeric donor because it is most homologous to β but forms heteromeric and not homomeric receptors [9]. Moreover, the θ ICD shares virtually no homology with the β_3_ ICD or any other subunit. Because there is no evidence for ICD involvement in assembly, we hypothesized that the θ ICD would support the functional expression of β_3_ as either homomers or heteromers. We used immunofluorescence staining to test plasma membrane expression and whole-cell patch-clamp with fast perfusion to test receptor function.

Results indicate that neither the β_3_/θ_IC_ nor the θ/β_3IC_ chimera can traffic to the plasma membrane when expressed alone, suggesting the β_3_ ICD is necessary for functional homomeric expression but is insufficient to traffic unassembled θ subunits to the plasma membrane. When expressed in combination with either wild-type α_3_ or β_3_, the β_3_/θ_IC_ chimera was rescued to the plasma membrane and the receptor functioned essentially like the wild type β-containing receptor, while the θ/β_3IC_ chimera was not rescued. This suggests that the TM3-TM4 intracellular loops of α_3_ and β_3_ each contain essential anterograde trafficking signals that are required to overcome ER retention of assembled homo- or heteropentamers.

## Results

### The IC loop of θ disrupts β_3_ homomeric plasma membrane expression

**Figure 1.**
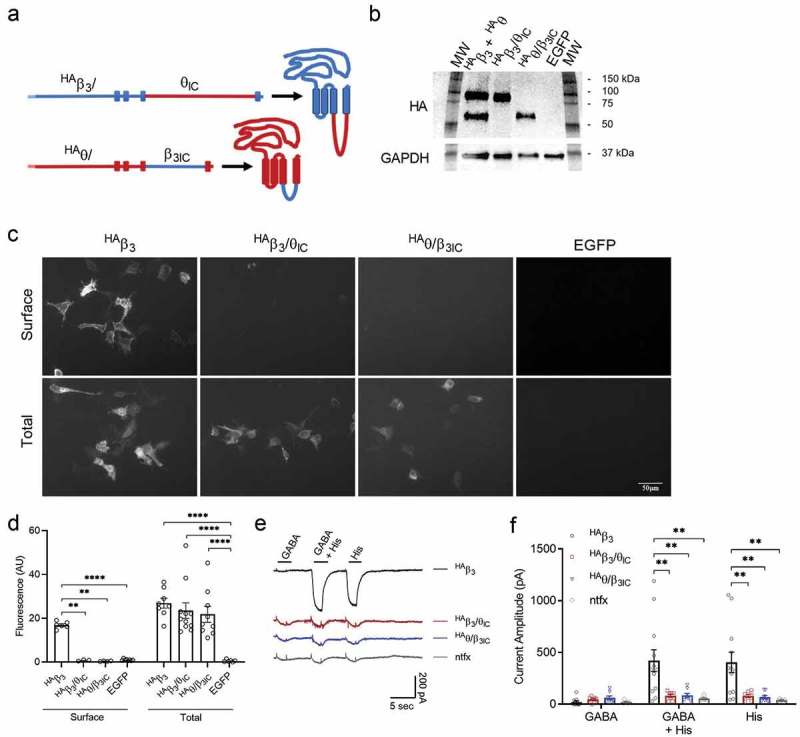
Homomeric expression and function of β_3_ and chimera. a) Schematic of the ^HA^β_3_/θ_IC_ and ^HA^θ/β_3IC_ chimeras where the blue portions are from β_3_ and the red portions are from θ. b) Total protein Western blot of ^HA^β_3_ + ^HA^θ, ^HA^β_3_/θ_IC_, ^HA^θ/β_3IC_, and EGFP transfected HEK293 cells. The membrane was probed with primary rabbit anti-HA Epitope Tag (1:5000 dilution) and rabbit anti-GAPDH (1:5000 dilution) and secondary goat anti-rabbit-HRP (1:5000 dilution). HA bands were quantified as fold-changes against GAPDH using ImageJ. Compared to GAPDH, the ratio of ^HA^β_3_ = 1.87; ^HA^θ = 1.36; ^HA^β_3_/θ_IC_ = 1.53; ^HA^θ/β_3IC_ = 1.61. c) Representative IF images at 20x magnification of non-permeabilized (surface) and permeabilized (total) staining of ^HA^β_3_, ^HA^β_3_/θ_IC_, ^HA^θ/β_3IC_, or EGFP expressed alone in HEK293 cells. Expression was determined using a rabbit anti-HA Epitope Tag DyLight^TM^ 549 conjugated antibody at 1:1000 dilution. d) Bar graphs portray the mean ± SEM of Fiji ImageJ fluorescence quantification of ^HA^β_3_ (n_surf_ = 6 images (46 cells), n_total_ = 8 images (79 cells)); ^HA^β_3_/θ_IC_ (n_surf_ = 4 images (0 cells), n_total_ = 11 images (97 cells)); and ^HA^θ/β_3IC_ (n_surf_ = 4 images (0 cells), n_total_ = 9 images (31 cells)) IF images from c with individual data points overlaid. e) Representative traces of ^HA^β_3_ (n = 12), ^HA^β_3_/θ_IC_ (n = 7/8), and ^HA^θ/β_3IC_ (n = 8/10) in response to 1 mM GABA and 3 mM histamine applied separately or together. f) Bar graphs portray the mean ± SEM of peak current amplitudes from whole-cell recordings in e with individual data points overlaid. * = p < 0.05; ** = p < 0.01; *** = p < 0.001; **** = p < 0.0001; using 2-way ANOVAs with Bonferroni post hoc comparisons.

To determine how the intracellular loop is involved in functional expression, we produced a reciprocal pair of chimeric receptors (β_3_/θ_IC_ and θ/β_3IC_) swapping the β_3_ and θ ICDs Figure 1(a). Unlike β_3_, θ subunits do not assemble or express as homomeric receptors on the plasma membrane [9]. To visualize expression, the wild-type β_3_ and both chimeric constructs were tagged with three consecutive hemagglutinin epitopes (HA) on the N-terminus between amino acids 5 and 6 (^HA^θ/β_3IC_) or 6 and 7 (^HA^β_3_, ^HA^β_3_/θ_IC_) of the mature protein. Western blotting analysis was performed to confirm that the chimeric constructs are full length and expressed with a similar relative abundance compared to either the ^HA^β_3_ or the ^HA^θ construct. As expected, ^HA^β_3_/θ_IC_ was found to be a similar molecular weight as ^HA^θ (^HA^β_3_/θ_IC_ calculated MW = 74 kDa, actual MW = 91 kDa and ^HA^θ calculated MW = 77 kDa, actual MW = 95 kDa). Likewise, ^HA^θ/β_3IC_ was found to be a similar molecular weight as ^HA^β_3_ (^HA^θ/β_3IC_ calculated MW = 61 kDa, actual MW = 63 kDa and ^HA^β_3_ calculated MW = 58 kDa, actual MW = 64 kDa). GAPDH was used as a loading control and had a molecular weight of 36 kDa, as expected. Relative to GAPDH, the expression ratio of ^HA^β_3_ was 1.87, ^HA^θ was 1.36, ^HA^β_3_/θ_IC_ was 1.53, and ^HA^θ/β_3IC_ was 1.61 Figure 1(b). Next, we used immunofluorescence to visualize and quantify subunit expression in non-permeabilized cells (surface) and after Triton-X permeabilization (total). When expressed alone in HEK293 cells, ^HA^β_3_ was clearly labeled on the surface of many cells, as shown by non-permeabilized staining with an anti-HA antibody Figure 1(c,d). In contrast, ^HA^β_3_/θ_IC_ was rarely found on the surface (max 1–5 cells per dish compared to ≥ 150 cells for ^HA^β_3_); but, when it was seen, the fluorescence intensity was comparable to ^HA^β_3_ (^HA^β_3_/θ_IC_ = 14.3 ± 2.8 AU, n = 10 images (10 cells) and ^HA^β_3_ = 16.9 ± 0.6 AU, n = 6 images (46 cells)). ^HA^θ/β_3IC_ was never found to be labeled on the surface of cells (n = 4 images (0 cells)). Total expression levels of the ^HA^β_3_, ^HA^β_3_/θ_IC_, and ^HA^θ/β_3IC_ constructs were comparable in terms staining intensity and cell number with the exception of ^HA^θ/β_3IC_, which showed fewer cells labeled (^HA^β_3_ = 26.9 ± 2.3 AU, n = 8 images (79 cells); ^HA^β_3_/θ_IC_ = 23.5 ± 3.6 AU, n = 11 images (97 cells); and ^HA^θ/β_3IC_ = 21.8 ± 3.7 AU, n = 9 images (31 cells)) when the cells were re-probed following permeabilization.

Functionally, β_3_ homomers are gated by histamine and only weakly, if at all, by GABA [10,11]. To better quantify functional expression levels, we used patch clamp recording with fast perfusion to measure whole cell currents in response to 1 mM GABA and 3 mM histamine applied separately or together Figure 1(e,f). As expected, ^HA^β_3_ homomeric receptors exhibited robust responses to both histamine and GABA + histamine in all cells recorded. ^HA^β_3_ homomeric receptors showed large histamine-evoked currents (404 ± 96 pA, n = 12), which were similar when GABA and histamine were co-applied (420 ± 101 pA, n = 12). GABA-evoked currents were comparatively small (21 ± 10 pA, n = 12) and unreliable. ^HA^β_3_/θ_IC_ had one non-responsive cell, resulting in 7 out of 8 recordings with measurable responses. From the responding cells, homomeric ^HA^β_3_/θ_IC_ chimeric receptors generated very small currents in response to histamine (70 ± 17 pA, n = 7/8) or GABA + histamine (74 ± 1 pA, n = 7/8), which were significantly smaller than ^HA^β_3_ currents (p = 0.006 and p = 0.003, respectively). ^HA^β_3_/θ_IC_ also generated very small GABA-evoked currents (43 ± 10 pA, n = 7/8), however these were not significantly different from ^HA^β_3_ currents (21 ± 10 pA, n = 12, p > 0.9) or currents from non-transfected cells (21 ± 6 pA, n = 6/16, p > 0.9). We also tested the reciprocal IC chimera, ^HA^θ/β_3IC_. In this case, 8 out of 10 recordings had measurable responses. Like ^HA^β_3_/θ_IC_, homomeric ^HA^θ/β_3IC_ generated very small currents in response to histamine (68 ± 15 pA, n = 8/10) and GABA + Histamine (86 ± 18 pA, n = 8/10), which were significantly smaller than ^HA^β_3_ (p = 0.002 for both comparisons). The GABA currents generated by ^HA^θ/β_3IC_ (62 ± 17 pA, n = 8/10) were also not significantly different from ^HA^β_3_ or non-transfected cells. Taken together, the immunofluorescence and functional results suggest that the β_3_ ICD is required for efficient homomeric surface expression but is not sufficient for surface expression in the absence of assembly.

## The α_3_ subunit rescues chimeric functional expression

**Figure 2.**
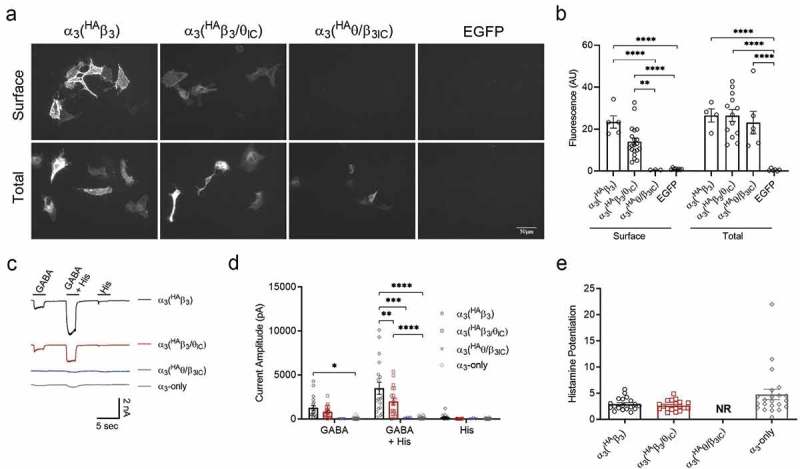
Heteromeric expression and function of β_3_ and chimera.a) IF image at 20x magnification of non-permeabilized (surface) and permeabilized (total) staining of ^HA^β_3_, ^HA^β_3_/θ_IC_, or ^HA^θ/β_3IC_ in combination with α_3_ co-expressed in HEK293 cells using EGFP as a negative control. Expression was determined using a rabbit anti-HA-549 antibody at 1:1000 dilution. b) Bar graphs portray the mean ± SEM of Fiji ImageJ fluorescence quantification of α_3_(^HA^β_3_) (n_surf_ = 5 images (82 cells), n_total_ = 4 images (60 cells)); α_3_(^HA^β_3_/θ_IC_) (n_surf_ = 20 images (100 cells), n_total_ = 13 images (121 cells)); and α_3_(^HA^θ/β_3IC_) (n_surf_ = 4 images (0 cells), n_total_ = 6 images (17 cells)) IF images from a with individual data points overlaid. c) Representative traces of α_3_(^HA^β_3_) (n = 19), α_3_(^HA^β_3_/θ_IC_) (n = 19/20), and α_3_(^HA^θ/β_3IC_) (n = 3/9) in response to 1 mM GABA and 3 mM histamine applied separately or together. d) Bar graphs portray the mean ± SEM of peak current amplitudes from whole-cell recordings in c with individual data points overlaid. e) Bar graphs portray the mean ± SEM of the degree of histamine potentiation with individual data points overlaid. * = p < 0.05; ** = p < 0.01; *** = p < 0.001; **** = p < 0.0001; using 2-way ANOVAs (b and d) or 1-way ANOVAs (e) with Bonferroni post hoc comparisons.

The reduction of β_3_ functional expression by the θ ICD raises the question of whether this is caused by an assembly or a trafficking defect. Because heteromeric assembly is more common among GABA_A_ receptors than homomeric assembly, we wanted to know if the θ ICD would also prevent the functional expression of αβ heteromeric receptors. To test this, we co-transfected the untagged α_3_ subunit with ^HA^β_3_, ^HA^β_3_/θ_IC_, or ^HA^θ/β_3IC_ in parallel cultures of HEK293 cells. α_3_(^HA^β_3_) and α_3_(^HA^β_3_/θ_IC_) showed clear plasma membrane labeling by anti-HA in non-permeabilized conditions Figure 2(a,b). There were similar numbers of surface-labeled cells and the fluorescence intensity trended toward a decrease in α_3_(^HA^β_3_/θ_IC_) compared to α_3_(^HA^β_3_) (14.0 ± 1.7 AU, n = 20 images (100 cells) and 23.4 ± 3.0 AU, n = 5 images (82 cells), respectively) but failed to reach significance (p = 0.07). α_3_(^HA^θ/β_3IC_) showed no anti-HA surface labeling (n = 4 images (0 cells)). Total HA expression was equivalent in all three conditions when the cells were re-probed following permeabilization (α_3_(^HA^β_3_) = 26.5 ± 6.2 AU, n = 4 images (60 cells); α_3_(^HA^β_3_/θ_IC_) = 26.5 ± 2.8 AU, n = 13 images (121 cells); and α_3_(^HA^θ/β_3IC_) = 23.1 ± 5.4 AU, n = 6 images (17 cells)).

Functionally, α_3_β_3_ heteromeric receptors are gated by GABA and not by histamine, but the GABA responses are strongly potentiated by histamine [10]. To better quantify functional expression levels, as before, we used patch clamp recording with fast perfusion to measure whole cell currents in response to 1 mM GABA and 3 mM histamine applied separately or together Figure 2(c,d). α_3_(^HA^β_3_) had measurable responses in all cells recorded, α_3_(^HA^β_3_/θ_IC_) had measurable responses in 19 out of 20 cells recorded, α_3_(^HA^θ/β_3IC_) had measurable responses in 3 out of 9 cells recorded, and the control, α_3_-only, had measurable responses in 23 out of 28 cells recorded. Comparing the peak amplitudes of GABA-evoked currents, there was no significant difference between α_3_(^HA^β_3_) and α_3_(^HA^β_3_/θ_IC_) (1266 ± 270 pA, n = 19 and 803 ± 155 pA, n = 19/20, respectively) or between α_3_(^HA^θ/β_3IC_) and α_3_-only (7 ± 6 pA, n = 3/9 and 66 ± 22 pA, n = 23/28, respectively). On average, the combined response to GABA + histamine was significantly smaller in both the α_3_(^HA^β_3_/θ_IC_) and α_3_(^HA^θ/β_3IC_) heteromeric conditions (1983 ± 363 pA, n = 19/20 and 108 ± 19, n = 3/9, respectively) compared to the α_3_(^HA^β_3_) heteromers (3483 ± 671 pA, n = 19) (α_3_(^HA^β_3_/θ_IC_) p = 0.002 and α_3_(^HA^θ/β_3IC_) p = 0.0001). The GABA + histamine response from α_3_(^HA^θ/β_3IC_) was not significantly different from the response from α_3_-only transfection conditions. There was no response to histamine alone in α_3_(^HA^β_3_/θ_IC_) (10 ± 6 pA, n = 19/20) or α_3_(^HA^θ/β_3IC_) (32 ± 26 pA, n = 3/9) and a comparatively small response, relative to GABA, in α_3_(^HA^β_3_) (192 ± 61 pA, n = 19), suggesting a small but measurable population of β_3_ homomers in the latter condition. For both α_3_(^HA^β_3_) and α_3_(^HA^β_3_/θ_IC_), the GABA + histamine response was markedly potentiated compared to GABA alone, which is typical of αβ heteromers. There was no difference in the extent of histamine potentiation of the GABA response between α_3_(^HA^β_3_) (2.93 ± 0.27-fold, n = 19), α_3_(^HA^β_3_/θ_IC_) (2.64 ± 0.22-fold, n = 19), and α_3_-only (4.78 ± 0.96-fold, n = 22). α_3_(^HA^θ/β_3IC_) only responded to GABA in one recording and thus, could not be included in the statistical analysis. Altogether, these data suggest that the θ ICD chimera functions like the wild-type β_3_ subunit, albeit with marginally lower heteromeric surface expression levels and no homomeric surface expression.

**Figure 3.**
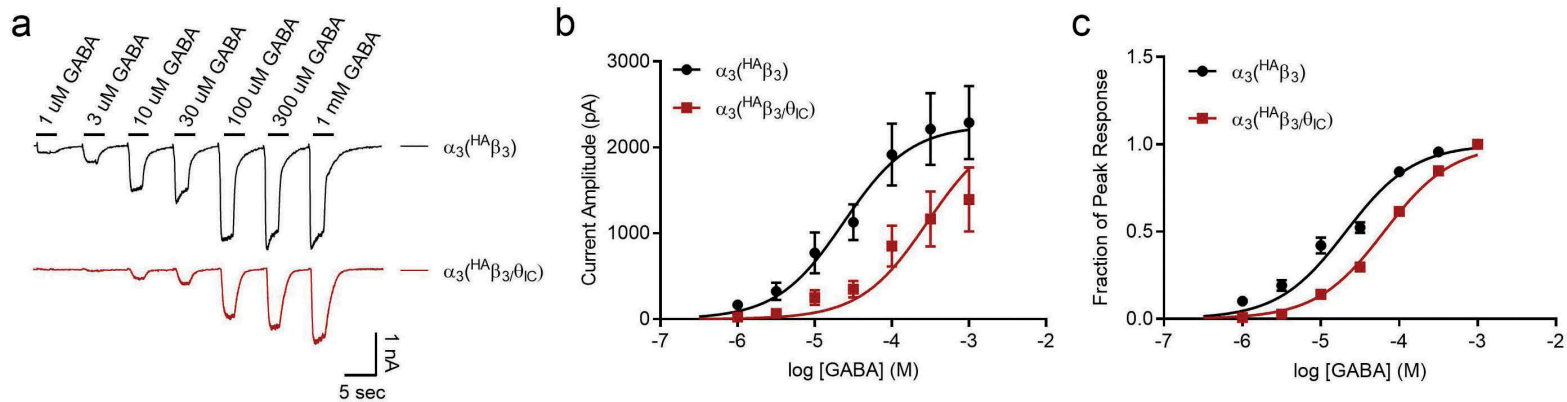
GABA concentration-response curves of αβ heteromeric combinations.a) Representative traces of GABA-evoked currents from α_3_(^HA^β_3_) (n = 10–20 cells per concentration) and α_3_(^HA^β_3_/θ_IC_) (n = 9–19 cells per concentration) in response to increasing GABA concentrations from 1 μM to 1 mM. b) Raw peak current amplitudes plotted as a function of GABA concentration. Fit parameters: α_3_(^HA^β_3_) EC_50_ = 28 μM, I_max_ = 2384 pA; α_3_(^HA^β_3_/θ_IC_) EC_50_ = 86 μM, I_max_ = 1509 pA. c) Normalized peak amplitudes from B are plotted as a function of GABA concentration. Fit parameters: α_3_(^HA^β_3_) EC_50_ = 21 μM; α_3_(^HA^β_3_/θ_IC_) EC_50_ = 64 μM.

To further explore any functional differences caused by the θ ICD, we compared agonist potencies in αβ heteromers containing either ^HA^β_3_ or ^HA^β_3_/θ_IC_ (Figure 3). ^HA^θ/β_3IC_ was not included in this experiment due to the lack of response seen in Figure 2. Concentration-response curves were constructed from the peak amplitudes of whole cell currents evoked sequentially by increasing concentrations of GABA (from 1 μM to 1 mM), as shown in Figure 3. Similar to the previous experiment, results showed a trend toward lower maximal peak currents from α_3_(^HA^β_3_/θ_IC_) heteromeric receptors (1509 ± 274.6 pA, n = 19) compared to α_3_(^HA^β_3_) heteromers (2384 ± 258 pA, n = 20); however, this trend failed to reach significance (p = 0.08; Figure 3(a,b). Concentration-response curves were normalized to the maximum GABA concentration tested, and the comparison of these curves revealed a rightward-shift to a 3-fold higher GABA EC_50_ for the chimera-containing heteromer. EC_50_ values were 21 μM (n = 10–20 cells per concentration) for α_3_(^HA^β_3_) and 64 μM (n = 9–19 cells per concentration) for α_3_(^HA^β_3_/θ_IC_) Figure 3(c). It is not clear if the 3-fold potency difference represents an effect of the θ ICD on β_3_ subunit function *per se* or a difference in αβ subunit stoichiometry between conditions, which could also explain the trend toward smaller current amplitudes.

## The β_3_ subunit also rescues chimeric functional expression

**Figure 4.**
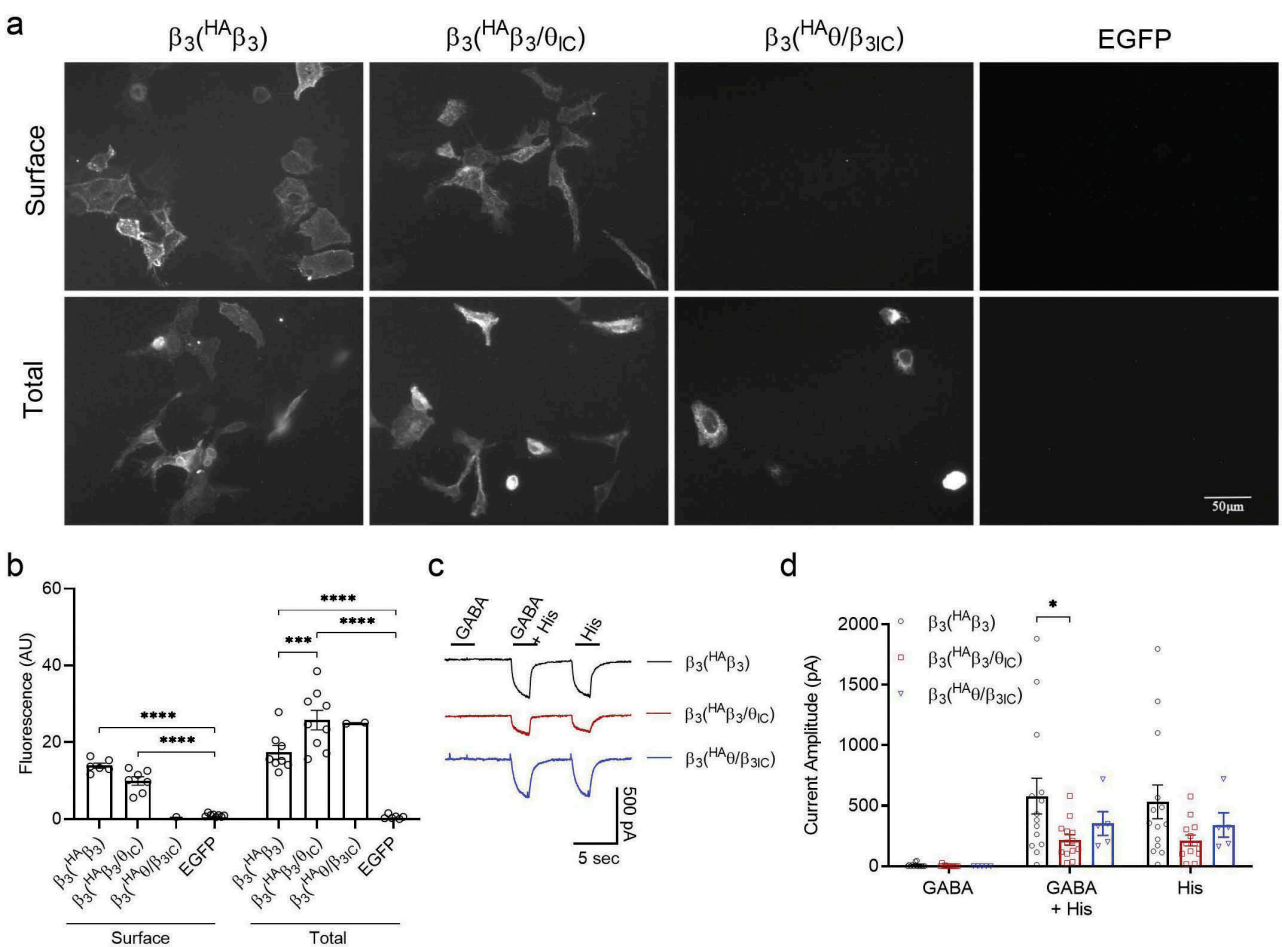
Pseudo-homomeric expression and function of wild type and chimeric β_3_.a) IF images at 20x magnification of non-permeabilized (surface) and permeabilized (total) staining of ^HA^β_3_, ^HA^β_3_/θ_IC_, or ^HA^θ/β_3IC_ in combination with β_3_ co-expressed in HEK293 cells using EGFP as a negative control. Expression was determined using a rabbit anti-HA-549 antibody at 1:1000 dilution. b) Bar graphs portray the mean ± SEM of Fiji ImageJ fluorescence quantification of β_3_(^HA^β_3_) (n_surf_ = 6 images (49 cells), n_total_ = 8 images (67 cells)); β_3_(^HA^β_3_/θ_IC_) (n_surf_ = 7 images (52 cells), n_total_ = 9 images (79 cells)); and β_3_(^HA^θ/β_3IC_) (n_surf_ = 2 images (0 cells), n_total_ = 2 images (7 cells)) IF images from a with individual data points overlaid. c) Representative traces of β_3_(^HA^β_3_) (n = 14), β_3_(^HA^β_3_/θ_IC_) (n = 13/15), and β_3_(^HA^θ/β_3IC_) (n = 5) in response to 1 mM GABA and 3 mM histamine applied separately or together. d) Bar graphs portray the mean ± SEM of peak current amplitudes from whole-cell recordings in c with individual data points overlaid. * = p < 0.05; ** = p < 0.01; *** = p < 0.001; **** = p < 0.0001; using 2-way ANOVAs with Bonferroni post hoc comparisons.

Our data clearly show that the ^HA^β_3_/θ_IC_ chimera is viable and readily assembles into functional surface-expressed receptors with α_3_. Since the known structural determinants for assembly are in the EC ligand binding domains and wild-type β_3_ readily assembles in a homomeric configuration, we reasoned that ^HA^β_3_/θ_IC_ probably also assembles as homomeric receptors but has a deficit in trafficking introduced by the θ ICD. Such deficit could either result from the loss of an anterograde trafficking signal contained in β ICD or the gain of an ER retention signal in the θ ICD that co-assembly with α_3_ can overcome. If so, the next logical question was whether co-assembly with wild-type β_3_, having its natural ICD, could also rescue the θ ICD chimera. To test this, we co-transfected the untagged β_3_ subunit with ^HA^β_3_, ^HA^β_3_/θ_IC_, or ^HA^θ/β_3IC_ in parallel cultures of HEK293 cells to produce pseudo-homomeric β_3_ receptors having either the β ICD on all subunits or a mixture of β and θ ICDs. Remarkably, when co-transfected with wild-type β_3_, clear surface labeling was seen for both ^HA^β_3_ and ^HA^β_3_/θ_IC_, as shown by anti-HA staining in non-permeabilized conditions Figure 4(a,b). Wild-type β_3_ co-expression with both ^HA^β_3_ and ^HA^β_3_/θ_IC_ yielded comparable numbers of surface-labeled cells with similar intensity (β_3_(^HA^β_3_) = 13.9 ± 0.7 AU, n = 6 images (49 cells) and β_3_(^HA^β_3_/θ_IC_) = 9.9 ± 1.1 AU, n = 7 images (52 cells)). Co-expression of wild-type β_3_ with ^HA^θ/β_3IC_ showed no surface labeling by anti-HA (n = 2 images (0 cells)). When the cells were re-probed following permeabilization there was a significantly higher fluorescence intensity but a similar number of labeled cells in β_3_(^HA^β_3_/θ_IC_) (25.8 ± 2.6 AU, n = 9 (79 cells)) compared to β_3_(^HA^β_3_) (17.4 ± 1.8 AU, n = 8 (67 cells)) (p = 0.0003). There were not enough fields containing labeled cells for β_3_(^HA^θ/β_3IC_) to be included in the statistical analysis (25.0 AU, n = 2 images (7 cells)).

Functionally, we tested the pseudo-homomeric combinations in the same manner as the homomeric receptors and the α_3_ heteromers Figure 4(c,d). Since the β_3_(^HA^β_3_) and β_3_(^HA^β_3_/θ_IC_) combinations both contain the β_3_ EC ligand binding and TM domains, the pseudo-homomers were expected to behave like β_3_ homomeric receptors and give histamine-evoked but not GABA-evoked currents. Because the β_3_(^HA^θ/β_3IC_) combination contains the both the β_3_ and θ EC ligand binding domains, and we would not expect the θ NTD to bind histamine, it was unclear how the heteromers should behave if they were produced. The β_3_(^HA^β_3_) and β_3_(^HA^θ/β_3IC_) responses were measurable in all cells recorded and the β_3_(^HA^β_3_/θ_IC_) response was measurable in 13 out of 15 cells. In all conditions, there was a comparable but minimal response to 1 mM GABA (β_3_(^HA^β_3_) = 8 ± 4 pA, n = 14; β_3_(^HA^β_3_/θ_IC_) = 3 ± 3 pA, n = 13/15; and β_3_(^HA^θ/β_3IC_) = 0 ± 0 pA, n = 5) and much larger currents evoked by 3 mM histamine (β_3_(^HA^β_3_) = 533 ± 123 pA, n = 14; β_3_(^HA^β_3_/θ_IC_) = 213 ± 43 pA, n = 13/15; and β_3_(^HA^θ/β_3IC_) = 342 ± 90 pA, n = 5). There were no significant differences in the GABA or histamine responses for the three conditions, however the histamine-evoked current from β_3_(^HA^β_3_/θ_IC_) trended toward a decrease compared to β_3_(^HA^β_3_) (p = 0.08). The currents elicited by GABA + histamine together were significantly different between the β_3_(^HA^β_3_) and β_3_(^HA^β_3_/θ_IC_) conditions (580 ± 129 pA, n = 14 and 220 ± 42 pA, n = 13/15, respectively, p = 0.03) while the currents from β_3_(^HA^θ/β_3IC_) were not significantly different from either β_3_(^HA^β_3_) or β_3_(^HA^β_3_/θ_IC_) currents (β_3_(^HA^θ/β_3IC_) = 353 ± 88 pA, n = 5). Taken together, the immunofluorescence and whole cell recordings suggest that the β_3_/θ_IC_ chimera is, indeed, surface-expressed in a functional complex with wild-type β_3_. However, the ^HA^θ/β_3IC_ chimera also gave histamine-evoked currents but no surface immunofluorescence, so we must also consider whether the functional responses are mostly or entirely generated by the wild-type subunits.

**Figure 5.**
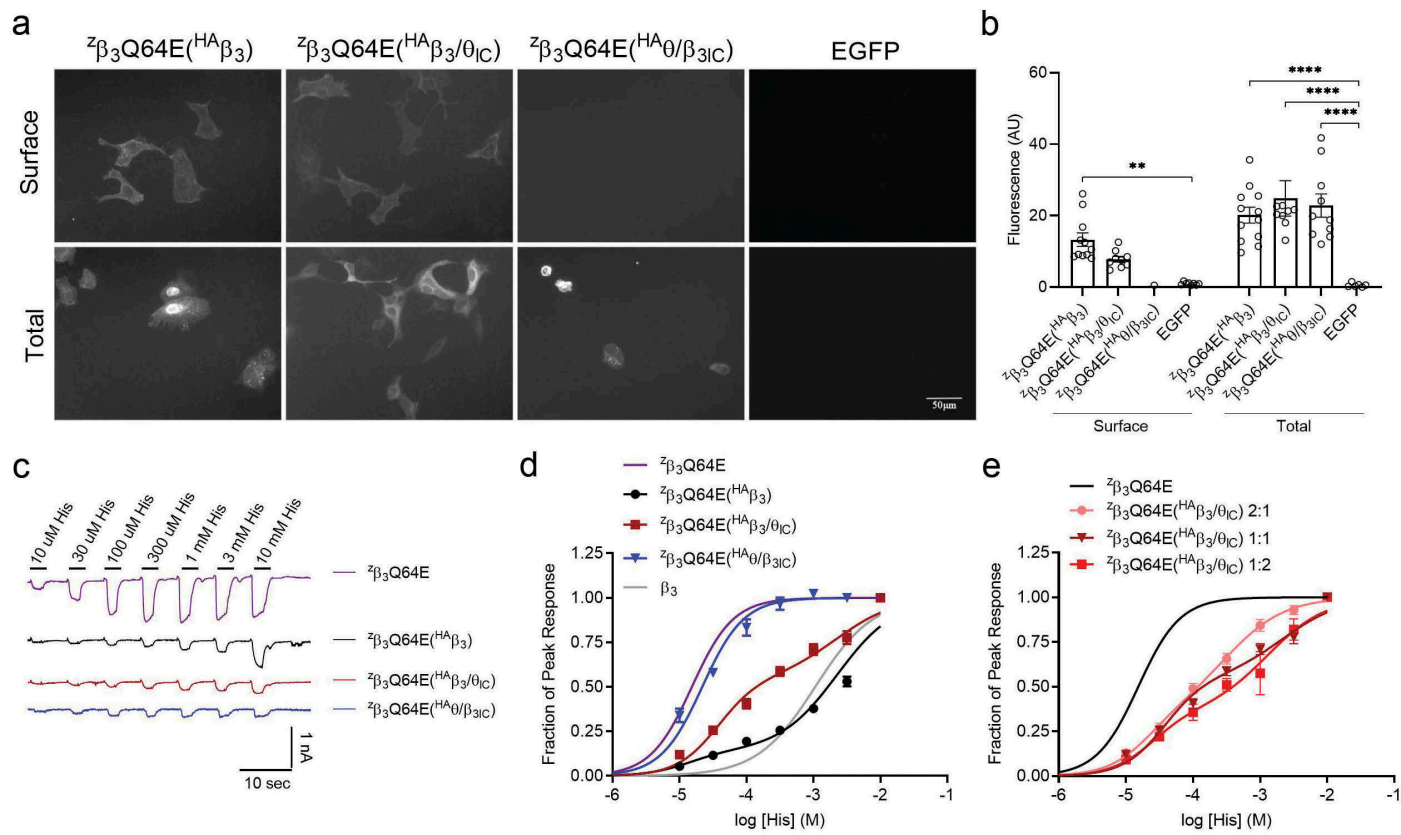
Histamine dose-response curves of pseudo-homomeric combinations.a) IF images at 20x magnification of non-permeabilized (surface) and permeabilized (total) staining of ^HA^β_3_, ^HA^β_3_/θ_IC_, or ^HA^θ/β_3IC_ in combination with ^z^β_3_Q64E co-expressed in HEK293 cells using EGFP as a negative control. Expression was determined using a rabbit anti-HA-549 antibody at 1:1000 dilution. b) Bar graphs portray the mean ± SEM of Fiji ImageJ fluorescence quantification of ^z^β_3_Q64E(^HA^β_3_) (n_surf_ = 11 images (46 cells), n_total_ = 12 images (65 cells)); ^z^β_3_Q64E(^HA^β_3_/θ_IC_) (n_surf_ = 9 images (26 cells), n_total_ = 9 images (51 cells)); and ^z^β_3_Q64E(^HA^θ/β_3IC_) (n_surf_ = 3 images (0 cells), n_total_ = 10 images (74 cells)) IF images from a with individual data points overlaid. c) Representative traces of ^z^β_3_Q64E (n = 8), ^z^β_3_Q64E(^HA^β_3_) (n = 12), ^z^β_3_Q64E(^HA^β_3_/θ_IC_) (n = 12), and ^z^β_3_Q64E(^HA^θ/β_3IC_) (n = 3) in response to increasing histamine concentrations from 10 μM to 10 mM. d) Normalized peak amplitudes from c plotted as a function of histamine concentration. Fit parameters: ^z^β_3_Q64E EC_50_ = 15 μM; ^z^β_3_Q64E(^HA^β_3_) EC_50_1 = 29 μM, EC_50_2 = 2.4 mM; ^z^β_3_Q64E(^HA^β_3_/θ_IC_) EC_50_1 = 39 μM, EC_50_2 = 1.3 mM; ^z^β_3_Q64E(^HA^θ/β_3IC_) EC_50_ = 22 μM; ^HA^β_3_ EC_50_ = 1.1 mM (n = 4–8 cells per concentration). e) Normalized peak amplitudes from combinations in C using varied cDNA ratios plotted as a function of histamine concentration. Fit parameters: ^z^β_3_Q64E(^HA^β_3_/θ_IC_) 2:1 ratio EC_50_1 = 21 μM, EC_50_2 = 330 μM (n = 7); ^z^β_3_Q64E(^HA^β_3_/θ_IC_) 1:2 ratio EC_50_1 = 25 μM, EC_50_2 = 1.3 mM (n = 6).

A functional tag that could distinguish the contributions of the individual subunits to overall receptor function was required to answer this question. We took advantage of a mutant β_3_ subunit, ^z^β_3_Q64E, which has a 50-fold higher affinity for histamine [11] and compared the histamine potencies at ^z^β_3_Q64E homomeric receptors and pseudo-homomeric receptors containing the ^z^β_3_Q64E mutant co-expressed with either ^HA^β_3_, ^HA^β_3_/θ_IC_, or ^HA^θ/β_3IC_ in parallel cultures of HEK293 cells. Concentration-response curves were constructed from whole cell current amplitudes evoked sequentially by increasing concentrations of histamine (from 10 μM to 10 mM), as shown in Figure 5. If the θ ICD-containing chimera is, in fact, a component of the functional surface receptor population in complex with the ^z^β_3_Q64E mutant, it would cause a rightward-shift in the concentration-response and the emergence of a biphasic curve because the chimera does not carry the high-affinity mutations. If it is not, then the functional receptors should behave like ^z^β_3_Q64E mutant homomers, having apparent high affinity for histamine and only one phase in the curve.

We first looked at non-permeabilized anti-HA staining to confirm that ^z^β_3_Q64E could traffic with ^HA^β_3_ and ^HA^β_3_/θ_IC_ to the surface like the wild-type α_3_ and β_3_ subunits before. Indeed, the HA-immunofluorescence surface staining showed cells labeled in both the ^z^β_3_Q64E(^HA^β_3_) and the ^z^β_3_Q64E(^HA^β_3_/θ_IC_) conditions Figure 5(a,b). Like the previous α and β combinations, the surface fluorescence intensity of cells expressing ^HA^β_3_/θ_IC_ was not significantly different compared to that of cells expressing ^HA^β_3_ (^z^β_3_Q64E(^HA^β_3_/θ_IC_) = 7.8 ± 0.8 AU, n = 9 images (26 cells); ^z^β_3_Q64E(^HA^β_3_) = 13.3 ± 1.9 AU, n = 11 images (46 cells)). Unsurprisingly, ^z^β_3_Q64E(^HA^θ/β_3IC_) showed no anti-HA surface labeling (n = 3 images (0 cells)). Permeabilized staining revealed similar numbers and fluorescence intensity of labeled cells (^z^β_3_Q64E(^HA^β_3_) = 20.2 ± 2.2 AU, n = 12 images (65 cells); ^z^β_3_Q64E(^HA^β_3_/θ_IC_) = 24.8 ± 4.9 AU, n = 9 images (51 cells); and ^z^β_3_Q64E(^HA^θ/β_3IC_) = 22.8 ± 3.6 AU, n = 8 images (74 cells)).

Figure 5 also shows that the concentration-response curves were right-shifted for both ^z^β_3_Q64E(^HA^β_3_) and ^z^β_3_Q64E(^HA^β_3_/θ_IC_) pseudo-homomeric combinations compared to ^z^β_3_Q64E alone, while the curve for ^z^β_3_Q64E(^HA^θ/β_3IC_) was not different from that of ^z^β_3_Q64E alone Figure 5(d). As shown previously in Hoerbelt et. al. (2016)[11], ^z^β_3_Q64E homomeric receptors have a very low Histamine EC_50_ (15 μM, n = 8) compared to the wild type β_3_ (1.1 mM, n = 4–8 cells per concentration). Comparing the maximal peak amplitudes evoked by 10 mM histamine, the whole cell currents from ^z^β_3_Q64E(^HA^β_3_/θ_IC_) and ^z^β_3_Q64E(^HA^θ/β_3IC_) pseudo-homomeric receptors (275 ± 55 pA, n = 12 and 178 ± 36 pA, n = 3, respectively) were smaller than either ^z^β_3_Q64E homomers (649 ± 201 pA, n = 8, p < 0.05) or the ^z^β_3_Q64E(^HA^β_3_) combination (804 ± 230 pA, n = 12, p < 0.05). Moreover, the concentration-response curves for the ^z^β_3_Q64E(^HA^β_3_) and the ^z^β_3_Q64E(^HA^β_3_/θ_IC_) combinations were clearly bimodal, having two components with markedly different concentration dependence. The histamine EC_50_ for homomeric ^z^β_3_Q64E was 15 μM (n = 8), which was similar to ^z^β_3_Q64E(^HA^θ/β_3IC_) (22 μM, n = 3) and the high-affinity component in both ^z^β_3_Q64E(^HA^β_3_) heteromers (16 μM, n = 12) and ^z^β_3_Q64E(^HA^β_3_/θ_IC_) heteromers (29 μM, n = 12). The low-affinity component for the for ^z^β_3_Q64E(^HA^β_3_) curve had an EC_50_ of 2.4 mM (n = 12) and represented 85% of the total curve fit, while the low-affinity component for the ^z^β_3_Q64E(^HA^β_3_/θ_IC_) curve had an EC_50_ of 1.3 mM (n = 8) and represented 53% of the total curve fit. These results confirm that the ^HA^β_3_/θ_IC_ chimera indeed assembles with other β_3_ subunits and contributes to the functional response, but to a slightly lesser extent than wild-type ^HA^β_3_ while the ^HA^θ/β_3IC_ chimera remains ER retained.

Figure 5(e) explores the effect of subunit ratios on shifting the concentration-response curve toward the high-affinity or low-affinity phenotypes. To this end, we compared ^z^β_3_Q64E(^HA^β_3_/θ_IC_) at 2:1, 1:1, and 1:2 ratios of cDNA. The maximal peak amplitudes evoked by 10 mM histamine were 754 ± 278 (n = 7), 275 ± 58 pA (n = 12), and 114 ± 22 pA (n = 6), respectively, consistent with a reduction in total surface receptors as the number of β ICDs was reduced. Regardless of the subunit ratio, the concentration-response curves retained their bimodality, suggesting that the HA-tagged constructs were assembled with the ^z^β_3_Q64E construct. The high-affinity component of the ^z^β_3_Q64E(^HA^β_3_/θ_IC_) curves at 2:1, 1:1, and 1:2 ratios had an EC_50_ of 21 μM (n = 7), 29 μM (n = 12), and 25 μM (n = 6), respectively, which are all comparable to the EC_50_ for ^z^β_3_Q64E (15 μM, n = 8). Similarly, the low-affinity component of the ^z^β_3_Q64E(^HA^β_3_/θ_IC_) curves at 1:2 and 1:1 ratios both had an EC_50_ of 1.3 mM (1:2 ratio n = 6, 1:1 ratio n = 12) which is comparable to the EC_50_ for wild-type β_3_ (1.1 mM, n = 4–8 cells per concentration). The low-affinity component accounted for 64% of the ^z^β_3_Q64E(^HA^β_3_/θ_IC_) curve at 1:2 ratio compared to 53% at 1:1. Interestingly, the low-affinity component was also 64% of the curve fit for ^z^β_3_Q64E(^HA^β_3_/θ_IC_) at 2:1 ratio, but the fit had an EC_50_ of 330 μM (n = 7), suggesting a difference in the number or functional impact of low-affinity sites present at the surface in this condition. The inverse relationship between maximal current amplitudes and the ^HA^β_3_/θ_IC_ content implies that multiple copies of the β_3_ ICD are probably required in the pentamer to overcome ER retention and traffic the receptor to the surface.

## Discussion

GABA_A_ receptors are functionally expressed on the cell surface. This requires translation by ER-associated ribosomes, the proper folding of the individual subunit proteins, their assembly into pentameric ion channels, and their trafficking through the secretory pathway to the plasma membrane where they can respond to extracellular signals. The cellular mechanisms that regulate folding, assembly and trafficking are not completely understood. Some of the more general maturation processes involve common ER chaperones like calnexin, BiP, and protein disulfide isomerase, which aid in folding, glycosylation, and formation of intra- or inter-subunit disulfide bonds[12]. Others may be more specific for particular subunits or combinations of subunits. Which subunits are compatible to assemble with one another, for example, appears to be determined by their extracellular ligand binding loops A-E and the GKER motif in loop F of the NTD [3,7,12]. Then, trafficking of the pentameric receptors from ER to the plasma membrane might occur by default but often appears to involve cytosolic chaperone proteins that interact with the cytoplasmic TM3-TM4 loops of different subunits to help guide and cluster the receptors to the appropriate location on the plasma membrane [13,14].

In the present study, we sought to test whether and how the ICD is involved in β_3_ homomeric and heteromeric functional expression. The β_3_ subunit can form homo-pentameric ion channels, unlike β_2_, and previous studies suggested this could be fully explained by differences in an “assembly motif” (GKER/DNTK) in Loop F of their NTD binding domains [7]. Up to now, the β ICD was not known to regulate recombinant functional expression. To explore the role of the ICD, we created reciprocal chimeric exchanges of the TM3-TM4 ICD between the β_3_ and θ subunits. We expected that the θ ICD would support the functional expression of chimeric β_3_/θ homomers. It did not. While the θ ICD did not disrupt assembly, our data revealed that the θ ICD could not support ER export and trafficking of receptors unless it was co-assembled with α or β subunits bearing their natural ICD. Of note, it is curious that the ^HA^θ/β_3IC_ chimera was not surface expressed in combination with α as previously reported for the wild-type θ subunit[9], but our data suggest they fail to co-assemble.

Results show that ^HA^β_3_ homomeric receptors can traffic to the plasma membrane while neither the ^HA^β_3_/θ_IC_ chimera nor the ^HA^θ/β_3IC_ chimera could do this. By contrast, we also show that pseudo-homomeric receptors composed of the ^HA^β_3_/θ_IC_ chimera plus wild-type β_3_ or the high-affinity ^z^β_3_ mutant are functionally expressed on the plasma membrane, as are heteromeric receptors composed of the ^HA^β_3_/θ_IC_ chimera plus wild-type α_3_. Taken together, three conclusions can be drawn from these results: (1) the β_3_ ICD is necessary for functional expression of the homomeric receptor, (2) the α_3_ ICD likewise promotes functional expression of the heteromeric receptor, and (3) the θ ICD contains neither permissive signals to promote surface expression nor inhibitory signals to prevent it. In the rare cases where homomeric ^HA^β_3_/θ_IC_ was seen to reach the plasma membrane, these too may be pseudo-homomers assembled with endogenous β_3_, which is expressed in HEK293 cell cultures at low levels in the culture [15] as a whole, or involve some other factor that varies from cell to cell and leads to a few positively stained cells. Based on the absence of ^HA^θ/β_3IC_ surface staining under any conditions, and the fact that θ does not assemble as a homomeric receptor [9], we can also conclude that the β_3_ ICD is not sufficient to drive surface expression if the subunits are not assembled. Although this study did not test other subunit ICDs, we propose that β_1-3_ all contain similar anterograde trafficking signals. This is supported by previous reports by other groups showing that homomeric β_1_ expression[3], and homomeric β_2_ mutants having the assembly-permissive GKER motif in their F-loop but still having the β_2_ ICD were also functionally expressed[7].

Virtually all neurons in the CNS express functional GABA_A_ receptors. Some of the 19 available subunits (e.g., α_1,_ β_2-3,_γ_2_) are expressed abundantly throughout the brain and are well characterized in native and recombinant systems[1]. Others are far less common (e.g., γ_1,_ γ_3,_ θ, ϵ, π) and are poorly understood[1]. Heterologous expression systems are often used in combination with mutagenesis to foster understanding of which subunits or combinations of subunits are functionally expressed, how they work and how they are regulated. HEK293 cells are by far the most commonly used mammalian cell line for the study of ligand-gated ion channels (LGIC), including GABA_A_ receptors, because they do not express LGIC subunits in an amount that would confound interpretation [15]. They are considered, in this regard, to be a blank slate. It is important, however, to consider that HEK293 cells are not neurons and, just as they generally do not express neuronal receptors, they also might not express all the proper machinery to process or traffic neuronal receptors in the same manner as neurons. This may be especially true for receptors or other cargo destined to axonal/presynaptic compartments, which HEK293 cells do not have.

Recent studies have made this point clearly with respect to nicotinic acetylcholine receptors (nAChR). The most widely expressed neuronal nAChRs, the α7 and α4β_2_ subtypes, have been notoriously difficult to study in mammalian cell lines because they are not functionally expressed [16–19]. However, co-expression of the putative chaperone/accessory proteins RIC-3 or TMEM35A/NACHO can permit robust functional expression of these nAChRs in HEK293 cells [17–21].

Our data indicate the ^HA^β_3_/θ_IC_ chimera alone rarely reaches the plasma membrane, whereas co-expression with either wild-type α_3_ or β_3_ rescues its surface expression. The defect in ^HA^β_3_/θ_IC_ homomeric expression appears to reflect a trafficking error, not an assembly error, because the pseudo-homomeric ^HA^β_3_/θ_IC_ plus wild-type β_3_ receptors are functionally expressed. From this we can infer that α_3_ and β_3_ subunits both contain anterograde trafficking signals that can overcome ER retention of the assembled receptors. In GABA_A_ subunits, and across all subunits of the pentameric ion channel superfamily, the TM3-TM4 intracellular loop is the most variable region both in terms of length and amino acid composition.

A number of cytosolic chaperones have been proposed to regulate GABA_A_ receptor trafficking by interactions involving the ICD of α, β or γ subunits. The first and best characterized chaperone shown to be involved in GABA_A_ receptor trafficking is the GABA_A_ receptor-associated protein, or GABARAP [22]. GABARAP acts by interacting with the γ_2_ ICD to promote plasma membrane expression [23]. Other cytosolic chaperones such as BIG2 (brefeldin A-inhibited GDP/GTP exchange factor 2), NSF (N-ethylmaleimide-sensitive factor), and PRIP1 (PLC-related catalytically inactive protein 1) all interact with the ICD of β_1-3_ [24–27]. Gephryn and PLIC1 (protein linking integrin-associated protein to cytoskeleton-1) interact with both α and β ICDs [14,28]. Except for PRIP1, these chaperone proteins and others are expressed at moderate to high levels in HEK293 cells (GEO accession number GDS5213) [29]. It remains to be determined how these proteins interact with the ICDs and which, if any, are required for functional expression of various subtypes of GABA_A_ receptors in HEK293 cells.

It is perhaps worth noting the relative homology among the synaptically expressed α, β, γ subunits, which are all readily expressed in heterologous systems. The rat β1-3 ICDs share 48–54% amino acid sequence identity, while the α1-3 and α5 ICDs are 36–62% identical, and the γ1-3 ICDs are 52–56% identical by comparing BLAST alignments. In contrast, the θ ICD has no homology in the rat genome and the non-synaptic α4, α6 and δ ICDs are likewise each unique. So, one question raised from this study remains: why do GABA_A_ β homomers and αβ heteromers express in HEK293 cells but the β/θ_IC_ chimera does not? If certain nAChRs are a precedent, it may be that the θ ICD requires interactions with a unique chaperone protein found only where θ is naturally expressed. If so, the β/θ_IC_ chimera could well express in another system, for example in neurons, or in heterologous cells upon co-expression of appropriate chaperones that are yet to be identified. Trafficking might also be amenable to proteostatic enhancement by small molecules [30]. As a whole, this study shows the involvement of the ICD in β_3_ homomeric and heteromeric functional expression and raises questions about the ICD of less common GABA_A_ subunits, such as θ. Further work is needed to determine precisely where the critical ICD motifs reside and to understand how the assembly and trafficking of β, θ, and the β/θ chimeric subunits are functionally regulated.

## Methods

### Drugs and solutions

Recording solution components, buffer components, GABA (cat. #A2129), histamine dihydrochloride (cat. #H7250) were purchased from Sigma-Aldrich (St. Louis, MO). GABA was dissolved in extracellular recording solution at a 1 M stock concentration, then stored at 4°C. Histamine was dissolved at a 30 mM stock concentration and the pH was adjusted to neutral. This stock histamine solution was made freshly or stored at −20°C for up to 72 hr. Stock drug solutions were diluted on the day of the experiment. Concentration-response curve solutions were made by serial dilution.

### cDNA and mutagenesis

All cDNA constructs were expressed in the pRK5 vector carrying the CMV promoter and ampicillin-resistance. Subcloning-efﬁciency E. coli were used as the host for cDNA copy replication. Rat α_3_ (accession no. L08492.1) and β_3_ (X15468.1) subunit cDNAs in the pRK5 vector were given generously by Dr. Peter Seeburg (Max Planck Institute for Medical Research, Heidelberg, Germany). Rat θ (AF419333.1) subunit cDNA was generously provided by Dr. Maurice Garrett (University of Bordeaux, Bordeaux, France) in the pcDNA3 vector and transferred into the pRK5 vector between BamHI (5ʹ) and XbaI (3ʹ) upon receipt. The coding region and UTRs were confirmed by Sanger sequencing for all subunit cDNAs and subsequent mutations. We identified two separate point mutations (causing H362N and L400M) within the ICD of our θ cDNA and one point mutation causing S296T in the third TMD of α_3_ that differ from the published sequences.

^HA^β_3_, ^HA^θ, ^HA^β_3_/θ_IC_, and ^HA^θ/β_3IC_ were all created using *NEBuilder HiFi DNA assembly kit* (New England Biolab, Ipswich, MA, E5520S). The hemagglutinin tag was a triplet of HA epitopes flanked by AgeI (5ʹ) and NotI (3ʹ) endonuclease sites. PCR primers were designed using the NEBuilder Assembly Tool v1.12. To make ^HA^β_3_, the triplet HA tag, TGLD**YPYDVPDYA**G**YPYDVPDYA**GS**YPYDVPDYA**AAA, was inserted between amino acids 6 and 7 of the mature β_3_ protein using the manufacturer’s protocol. In short, PCR was used to isolate and amplify the fragments of interest (linearized β_3_ and the HA tag). The fragments were then assembled by mixing and incubating at 50°C with the NEBuilder HiFi DNA Assembly Master Mix for ≥ 15min. The assembled product was then transformed into NEB5α competent cells and spread onto ampicillin-resistant plates, clonal colonies were picked and screened by endonuclease digestion. The ^HA^θ/pRK5 construct was made in the same manner as ^HA^β_3_/pRK5 except that the HA tag was inserted between amino acids 5 and 6 of the mature protein.

The ^HA^β_3_/θ_IC_ construct was made by linearizing ^HA^β_3_/pRK5 without the M3-M4 loop (IVFPFT … YIFFGR) and isolating the θ M3-M4 loop (RNHRRC … VPKVDR) from ^HA^θ/pRK5 using PCR. Likewise, to make the ^HA^θ/β_3IC_ construct, ^HA^θ/pRK5 was linearized (LFPLSF … YLFFSQ) and the β_3_ M3-M4 loop (QRQKKL … AIDRWS) was isolated from ^HA^β_3_/pRK5 using PCR. Fragments for both chimeras were then assembled and screened in the same manner as above.

The ^z^β_3_Q64E construct was made previously and described in Hoerbelt et. al. (2016).

### Cell culture and transfections

HEK293 cells (ATCC CRL-1573) were cultured at 37°C with 5% CO2 in Minimal Essential Medium plus glutamine (MEM, Gibco, Gaithersburg, MD) with 10% fetal bovine serum (Gibco) and 5% Penicillin-Streptomycin (Gibco) added. For immunofluorescence experiments and electrophysiological recording, cells were plated at 100,000/dish in Poly-D-Lysine (Sigma-Aldrich) coated 6-well plates (Corning) or 35 mm Nunc dishes (Nalge Nunc, Naperville, IL), left overnight to adhere, and then transfected with a total of the following per dish/well: 1 μg total cDNA (subunit ratios of 1:1 for co-transfections, unless otherwise stated), 0.82 μL Lipofectamine 2000 (Invitrogen), and 100 μL total serum-free MEM. Components were mixed using the manufacturer’s protocol. 100 μL of the transfection mixture was dripped in an outward spiraling motion into each dish or well. For Western blotting experiments, non-coated 60 mm dishes were used and cell density and transfection volumes were scaled up by a ratio of 0.4 due to the increase in surface area of the dish. Cells were used 36–40 hr post-transfection for immunofluorescence and Western blotting experiments and 20–48 hr post-transfection for electrophysiological recording. For electrophysiology transfections, EGFP/pRK5 was co-transfected with the GABA_A_ subunits as 10% of the total cDNA, and only cells expressing the EGFP were targeted to patch.

### Western blot

At 36–40h post transfection, HEK293 cells plated in 60 mm dishes were washed twice by PBS then lysed in 2% SDS + 8 mM EDTA. A fraction of the lysates was saved for a BCA analysis while the remaining fraction of the lysates was diluted 1:1 with reducing Laemmeli buffer (with 5% β-Mercaptoethanol). 10–20 μg of total protein was separated using a 10% SDS-PAGE gel. Separated proteins were then transferred to a nitrocellulose membrane. The membranes were washed with tris-buffered saline (pH = 7.4) + 0.1% Tween-20 (TBST) and then blocked with 5% milk in TBST. Blocked membranes were incubated at 4°C overnight in primary rabbit anti-HA Epitope Tag (1 mg/ml, diluted 1:5000 in 5% milk + TBST, Rockland Antibodies & Assays cat. #600-401-384). After washing thoroughly, the membranes were probed with secondary HRP-conjugated goat anti-rabbit IgG (1 mg/ml, diluted 1:5000 in 5% milk + TBST, Invitrogen cat. #ABIN964977) for 1 h at room temperature. Immunoreactivity was visualized using Pierce^TM^ ECL Western Blotting Substrate (ThermoFisher Scientific cat. #32,106) in a ChemiDoc Imaging System (BioRad, Hercules, CA, USA). Membranes were then incubated with primary rabbit anti-GAPDH (1 mg/ml, diluted 1:5000 in 5% milk + TBST, Sigma-Aldrich #G9545) for 1 h at room temperature. After washing well, the membranes were probed with the same secondary and visualized again as above. Band intensities and molecular weights were quantified using the ImageJ (NIH) gel analysis tool[31]. Blot images were post processed on a personal computer using Photoshop software for presentation.

### Immunofluorescence (IF)

At 36–40h post transfection, HEK293 cells plated in 6-well plates were washed in sucrose solution (290 mM sucrose, 5 mM HEPES, 3 mM KCl, 1.8 mM CaCl_2_, and 1 mM MgCl_2_; pH 7.3), then cells were washed in tris-buffered saline (TBS, pH 7.4), and fixed in TBS + 3.7% formaldehyde (pH 7.4). After fixation, cells were washed in TBS, blocked in TBS + 5% goat serum, then incubated for 1 hr in rabbit anti-HA Epitope Tag DyLight^TM^ 549 conjugated antibody (1 mg/ml, diluted 1:1000 in TBS + 5% goat serum, Rockland Antibodies & Assays #600-442-384). After washing with TBS, cells were visualized on an Olympus IX71 fluorescence microscope fitted with a LUCPlanFl 20X/0.4 RC2 (∞/) objective. Non-permeabilized fluorescent photomicrographs were captured at the same exposure with a QImaging QICAM digital camera (1X). After capturing non-permeabilized photomicrographs, the cells were then permeabilized in TBS + 0.1% TritonX-100. After permeabilization, cells were washed with TBS, blocked in TBS + 5% goat serum, re-incubated for 1 hr in the same antibody as above, and washed again with TBS. Cells were then visualized, and fluorescent photomicrographs of permeabilized staining were captured, as above. Both non-permeabilized (surface) and permeabilized (total) photomicrographs were post processed on a personal computer using Photoshop software for presentation.

### Immunofluorescence quantification

Average fluorescent intensities were calculated using Fiji ImageJ [31,32]. Using the 192 immunofluorescence photomicrographs captured as above, the fluorescent cells were manually outlined in each image, then the average fluorescent intensity was calculated and the background fluorescence was subtracted. The fluorescent intensities of immune-positive cells were reported in arbitrary units (AU).

### Electrophysiological recordings

Whole-cell patch clamp was used to examine the function of GABA_A_ receptors as in Fleck (2002) and Hoerbelt et al. (2015). Brieﬂy, at 20–48 hr post-transfection HEK293 cells were superfused with extracellular recording solution (pH 7.3–7.4; 295–305 mOsm) consisting of 0.1 mg/ml phenol red pH indicator and (in mM): 145 NaCl, 5 HEPES, 3 KCl, 1.8 CaCl_2_ and 1 MgCl_2_. Thin-walled borosilicate glass microelectrodes were 3–7MΩ when ﬁlled with intracellular recording solution (pH 7.3; 295–305 mOsm) consisting of (in mM): 135 CsCl, 10 CsF, 10 HEPES, 5 EGTA, 1 MgCl2 and 0.5 CaCl_2_. Patch recordings were conducted in voltage-clamp mode at −80 mV, causing inward chloride currents with V_rev_ around 0 mV under these ionic conditions. Current signals were recorded and analyzed on a Macintosh computer using Synapse software (Synergistic Research Systems, Silver Spring, MD). Drugs were applied mostly as described in Fleck (2002) and Hoerbelt et al. (2015). Brieﬂy, 4 or 8 syringes were loaded with extracellular recording solution with and without GABA and/or histamine and driven at 1.5 ml/min through a single glass ﬂow-pipe combining these 4 or 8 barrels. The ﬂow pipe had a ~200 mm diameter tip and was placed <1 mm from the target cell to allow complete superfusion of the cell. Rapid solution exchange (5–20ms) was provided by 3-way solenoid valves (Lee Co., Westbrook, CT) controlled by the computer. The protocols used for drug application were consistently 2 sec drug application pulses with at least 5 sec control solution application between each drug pulse.

### Data analysis and statistics

Current traces were analyzed with Synapse software (Silver Spring, MD), Kaleidagraph (Synergy Software, Reading, PA), GraphPad Prism 8 (San Diego, CA), and Microsoft Excel. All comparisons were made using cells transfected in parallel and recording dishes were alternated by transfection subtype. Current amplitudes were measured from baseline to peak, where the baseline was taken during control period immediately before the switch to drug application. Cells with noisy or unstable baselines were excluded from analysis. Histamine potentiation was assessed by comparing the current amplitude during combined application of GABA + histamine (or the extent of potentiation) to the current amplitude of the initial pulse of GABA alone. Concentration response curves were ﬁt using the following equation:

$$I_{\left[agonist\right]}=\frac{I_{max}}{1+{\left(\frac{EC_{50}}{\left[agonist\right]}\right)}^{n_{H}}}$$

Where $I_{max}$ = maximum current, $EC_{50}$ = concentration at half maximal current, and $n_{H}$ = Hill coefﬁcient. Current amplitudes for concentration response curves were normalized to the highest concentration of agonist tested then averaged. Biphasic histamine concentration response curves were fit using the following equation:

$$I_{\left[agonist\right]}=\frac{I_{HA}}{1+{\left(\frac{EC_{50}1}{\left[agonist\right]}\right)}^{n_{H1}}}+\frac{100-I_{HA}}{1+{\left(\frac{EC_{50}2}{\left[agonist\right]}\right)}^{n_{H2}}}$$

Where $I_{HA}$ = maximum high affinity current,$\;n_{H1}$ = Hill coefficient of the high affinity component (constrained to 1.4 based on the control fit to ^z^β_3_Q64E), $EC_{50}1$ = concentration at half maximal current of the high affinity component, $n_{H2}$ = Hill coefficient of the low affinity component (constrained to 1.0 based on the control fit to wild type β_3_), and $EC_{50}2$ = concentration at half maximal current of the low affinity component. Notably, there is a ~3 mV drop in Cl^−^ driving force at the highest histamine concentration tested (10 mM histamine dihydrochloride) which was not compensated. Half maximal (EC_50_) values from multiple replicates per group are presented as the mean ± the 95% conﬁdence interval. Other data are shown as mean ± SEM and n values refer to either the number of images analyzed followed by the total number of cells in all images acquired in parentheses or the number of cells recorded. For all statistical comparisons, a p value < 0.05 is considered statistically signiﬁcant.

## Data Availability

All data generated or analyzed during this study are included in this published article. Datasets analyzed are available from NCBI, accession number GDS5213.
